# Disambiguating “Mechanisms” in Pharmacy: Lessons from Mechanist Philosophy of Science

**DOI:** 10.3390/ijerph17061833

**Published:** 2020-03-12

**Authors:** Ahmad Yaman Abdin, Claus Jacob, Lena Kästner

**Affiliations:** 1 Division of Bioorganic Chemistry, School of Pharmacy, Saarland University, D-66123 Saarbruecken, Germany; s8ahabdi@stud.uni-saarland.de (A.Y.A.); c.jacob@mx.uni-saarland.de (C.J.); 2 Department of Philosophy, Saarland University, D-66123 Saarbruecken, Germany

**Keywords:** arrows, Baumkuchen model, causation, mode of action, multi-level mechanisms, new mechanist philosophy, pharmacodynamics

## Abstract

Talk of mechanisms is ubiquitous in the natural sciences. Interdisciplinary fields such as biochemistry and pharmacy frequently discuss mechanisms with the assistance of diagrams. Such diagrams usually depict entities as structures or boxes and activities or interactions as arrows. While some of these arrows may indicate causal or componential relations, others may represent temporal or operational orders. Importantly, what kind of relation an arrow represents may not only vary with context but also be underdetermined by empirical data. In this manuscript, we investigate how an analysis of pharmacological mechanisms in terms of producing and underlying mechanisms—as discussed in the contemporary philosophy of science—may shed light on these issues. Specifically, we shall argue that while pharmacokinetic mechanisms usually describe causal chains of production, pharmacodynamics tends to focus on mechanisms of action underlying the in vivo effects of a drug. Considering the action of thyroid gland hormones in the human body as a case study, we further demonstrate that pharmacodynamic schemes tend to incorporate entities and interactions on multiple levels. Yet, traditional pharmacodynamic schemes are sketched “flat”, i.e., non-hierarchically. We suggest that transforming flat pharmacodynamic schemes into mechanistic multi-level representations may assist in disentangling the different kinds of mechanisms and relations depicted by arrows in flat schemes. The resulting Baumkuchen model provides a powerful and practical alternative to traditional flat schemes, as it explicates the relevant mechanisms and relations more clearly. On a more general note, our discussion demonstrates how pharmacology and related disciplines may benefit from applying concepts from the new mechanist philosophy to guide the interpretation of scientific diagrams.

## 1. Introduction

Natural sciences in general, and biochemistry and pharmacy in particular, tend to investigate complex actions and interactions of and between entities, such as small-molecule compounds, biopolymers, cells and organisms. Some of these interactions can be assigned to the “Molecular Level”, where atoms, molecules, light and other forces interact. Others subsequently transcend to “higher levels” or scales, such as entire organisms in biology and medicine. At the highest levels, we even find interactions between organisms or between organisms and their environment as studied by psychology and sociology. (We are using the notions “level” and “layer” metaphorically here. Whether we should individuate these notions by disciplines, perspectives, scales, or some other means is not an issue we aim to delve into for now [1,2,3]. For the present purposes, all we need is the intuitive change in scale or domain, when we consider molecular vs. biological vs. social processes or objects.) Indeed, pharmacists take great pride in the fact that they are seemingly able to explain how molecules—be it natural products or chemically synthesized drugs—interact with biomolecules to produce physiological or even psychological changes. The processes relevant to such “higher-level” effects of “lower-level” processes are often described in terms of so-called “mechanisms”. These mechanisms are depicted in diagrams or schemes (we are using these notions interchangeably) representing certain entities (and on occasion, non-entities) such as structures and boxes, and activities or interactions as arrows. Such schemes are often referred to by scientists as “mechanisms”, without any further explanation.

**Figure 1:**
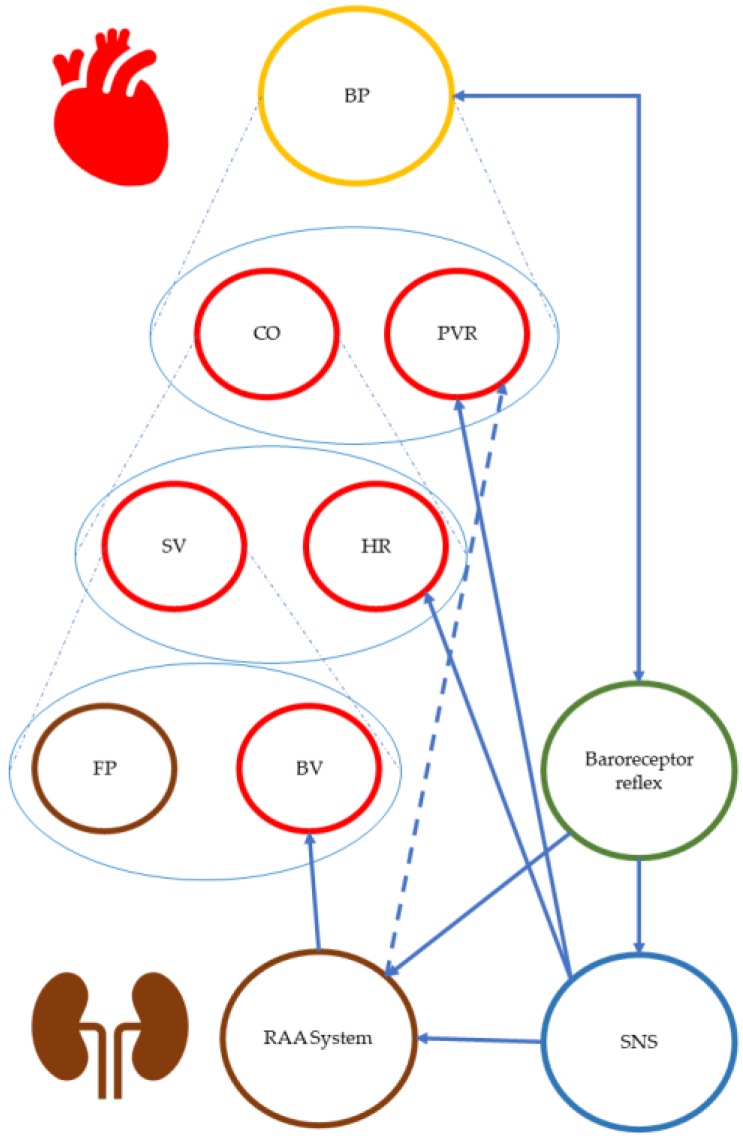
Schematic representation of the “mechanisms” regulating the blood pressure in the human body. BP: blood pressure, CO: cardiac output, PVR: peripheral vascular resistance, SV: stroke volume, HR: heart rate, BV: blood volume, FP: filling pressure (kidney), BV: blood volume, SNS: sympathetic nervous system, RAA System: renin-angiotensin-aldosterone system. This scheme has been adapted from a pharmacology textbook namely, Brenner and Stevens’ Pharmacology 5th edition p. 105) [4,5].

To illustrate this mechanistic approach common in the natural sciences, a rather basic example of such a mechanistic diagram is shown in Figure 1. This diagram depicts how different control and feedback loops within the human body regulate blood pressure. Here, the arrows connect various aspects of blood pressure and its regulatory systems, from blood volume and the heart to the hormones produced in various parts of the kidney and circulating in the blood stream.

Although such schemes are very attractive, it is unclear what exactly they convey. In order to address this question, the discussion in this manuscript draws on contemporary philosophy of science. We shall argue that (i) complex diagrams in pharmacology, such as the one shown above, superimpose representations of different types of mechanisms onto one another, and that (ii) arrows in these diagrams represent a number of different relations [3,6]. Indeed, it is clear to an expert that some of these arrows are intended to indicate causation (e.g., the arrows connecting the renin-angiotensin-aldosterone system (RAA) system with blood volume or the arrow between the sympathetic nervous system and the heart rate). For others, however, it is unclear what kind of relation they are intended to depict (e.g., the arrow from the RAA system to peripheral vascular resistance (PVR)). This lack of clarity may be resolved by examining diagrams with tools of the new mechanist philosophy [7,8]. Section 2 will briefly introduce mechanisms as they are typically discussed in contemporary philosophy of science. The focus then turns to pharmacy, in Section 3, to investigate in which sense and to which extent so-called “mechanisms of action” resemble mechanisms as they are discussed in philosophy. Our analysis will demonstrate that at least two kinds of “mechanisms of action” (MoA) must be distinguished: pharmacokinetic and pharmacodynamic ones. Curiously, this dichotomy between mechanisms in pharmacokinetic and pharmacodynamic resembles a distinction familiar within the philosophical debate: the distinction between productive causal and underlying constitutive mechanisms. The implications of this observation shall be discussed in Section 3.

Section 4 discusses arrows in pharmacological diagrams. Arrows are a central feature in the representation of complex pharmacological mechanisms. Given that these representations superimpose different kinds of mechanisms, one must wonder which—metaphysical—relations exactly each arrow represents. As a case study, the (bio-)synthesis of the thyroid gland hormones and their actions in the human body shall be considered. Our case study will highlight that certain arrows denote causal connections, while others represent temporal or operational orders. Yet, other arrows seem to function as a shorthand for entire mechanisms.

Section 5 will show how disentangling these different kinds of mechanisms may help clarify which kind of relation an arrow represents in a given pharmacological “box and arrow” diagram. An alternative to the traditionally “flat” schematic representations and inspired by mechanist philosophy will be introduced: the Baumkuchen (Spite Cake) model. This model strongly resembles a multi-level mechanism [6,7]. Note that the authors do not mean to commit to the notion that interlevel relations in Baumkuchen diagrams strictly conform to mechanist definitions of interlevel relations as local part-whole relations. Different Baumkuchen models might be created in light of the empirical underdetermination of certain relations. Thus, multi-level mechanistic analyses of mechanisms may not yield any strong metaphysical conclusion. Nonetheless, the discussion in this manuscript highlights that applying philosophical concepts of different mechanisms may assist pharmacists to better understand the mechanisms they work with, as well as the different kinds of arrows they use to represent them. Finally, Section 6 concludes that once disambiguated, the poised arrow in conventional “box and arrow” diagrams needs not be feared.

## 2. New Mechanist Philosophy: Underlying, Producing, and Maintaining Mechanisms

Recent discussions about explanations in the philosophy of science capitalize on the potential of mechanisms to provide integrated multi-level explanations [3,7,8]. According to the new mechanistic approach, scientists—especially in the Life Sciences—explain phenomena by discovering the mechanisms responsible for them. A range of different characterizations of mechanisms has been offered, but a general consensus may be expressed as follows [3,8,9,10]:

A mechanism for a phenomenon consists of entities (or parts) whose activities and interactions are organized such that they are responsible for the phenomenon to be explained.

This unifying characterisation usefully communicates the central tenets of the new mechanist philosophy. Still, it leaves important issues underspecified. For instance, there is considerable discussion about what exactly “phenomena” are [11,12,13]. For the current purposes, we shall simply take phenomena to be the explananda of mechanistic explanations, i.e., phenomena are what a mechanistic explanation seeks to explain. We assume that mechanistic explanations can serve to explain phenomena from various scientific domains. Accordingly, the explananda of mechanistic explanations may range from chemical reactions to cognitive capacities. Another, more pressing question is what it means for a mechanism to be “responsible for” a phenomenon [6,8,14,15]. We shall elaborate on this latter question below.

### 2.1. Three Kinds of Mechanisms

**Figure 2:**
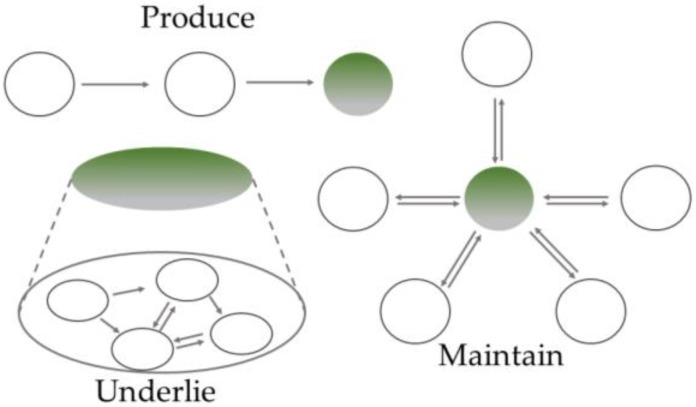
Three kinds of mechanisms; green circles depict the phenomenon to be explained (adapted from [3], p. 66) [3].

In their book on mechanism discovery, Craver and Darden suggest that there are three different kinds of mechanisms [3]: mechanisms which (i) produce, (ii) underlie, and (iii) maintain their phenomena, as depicted in Figure 2. Note that Craver and Darden talk about different *kinds of mechanisms*. While some may be inclined to read this as a metaphysical thesis, we prefer to consider this as a triad of three *kinds of mechanistic explanations*. If this is correct, being “responsible for” can take at least three different forms in mechanistic explanations. Firstly, it can refer to a causal relation where the phenomenon is the effect of the preceding mechanism’s operation. Secondly, “being responsible for” can denote a constitutive relation where the phenomenon is the overall behaviour of the mechanism (i.e., its organized parts, their activities and interactions). Lastly, it can describe some set of regulatory relations which keep a certain state stable or continuous process going. Accordingly, the mechanisms which produce, underlie, or maintain their phenomena are suited to explain different kinds of phenomena, i.e., to explain how a product or an end-result is generated, scientists will usually search for a mechanism which produced it; to explain a process, they will typically search for the mechanism underlying it; and to explain how a system’s stable state or continuous behaviour is maintained, they search for the mechanism maintaining it [6]. The idea that different kinds or types of explanations serve to answer different research questions is not new. In fact, many philosophers of science have assumed this [16,17,18]. Besides, this assumption is also inherent in the mechanistic view: mechanistic explanations are always mechanistic explanations *for* a specific phenomenon.

Our project in this manuscript is to examine how different kinds of mechanistic explanations act in concert in pharmacology. For the current purposes, we shall focus on producing and underlying mechanisms. An elaborate discussion of maintaining mechanisms demands for a project of its own.

### 2.2. Producing and Underlying

The distinction between producing and underlying mechanisms mirrors the familiar distinctions between etiological and constitutive explanations [18]. Still, there is more to this distinction than assuming a different phenomenon–mechanism relation: explanations describing producing and underlying mechanisms, respectively, have different explananda, i.e., end-products or overall processes. With this in mind, there are two straightforward rationalizations/descriptions as to how underlying and producing mechanisms relate (see [6] for a third plausible story).

**Figure 3:**
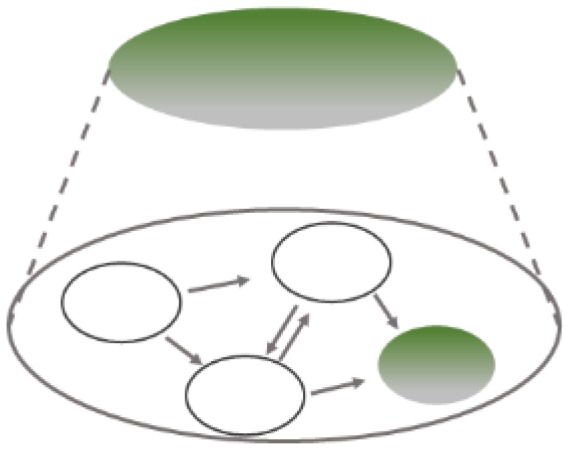
Production within an underlying mechanism. Please note that any component of the underlying mechanism shown in white may itself also be an explananda of another, even deeper underlying mechanism.

Firstly, a producing mechanism may be located within an underlying mechanism (see Figure 3). In studying the productive aspects within an underlying mechanism, scientists temporarily switch the explanandum: they focus on how a certain state or activity of a given component within the mechanism—shown in green—is produced.

**Figure 4:**
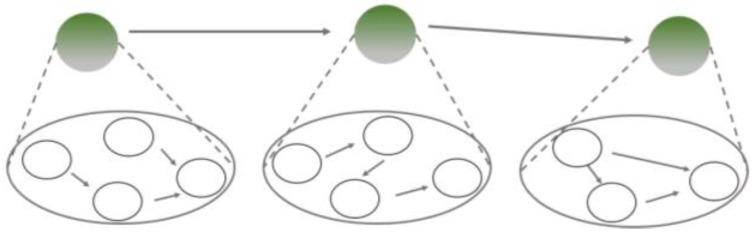
Mechanisms underlying components of a productive mechanism.

Secondly, each step in the causal sequence of a producing mechanism may be spelled out further by identifying the underlying mechanisms at each stage, the top circles shaded in green in Figure 4. As in the first case, scientists here change the explanandum. To investigate the underlying mechanisms at each stage, i.e., “phenomenon to be explained”, they must ask how each of the processes occurring within a productive chain is implemented rather than what is produced at the end of the sequence—thus, all of the top circles are shaded in green.

The major difference between these scenarios is how the information is integrated into a coherent picture. The first scenario provides an analysis of some of the causally productive processes within a mechanism recognized to underlie a phenomenon (Figure 3). By contrast, the second scenario spells out how the contributing processes in productive mechanisms are themselves mechanistically implemented, i.e., which mechanisms underlie them (Figure 4).

In Section 4 and Section 5, we shall demonstrate that in pharmacology and related disciplines, underlying and producing mechanisms are often superimposed on a single “box and arrow” representation of a mechanism of action (MoA). An analysis in terms of different kinds of mechanisms may usefully guide the interpretation of such traditionally “flat”, i.e., non-hierarchical, diagrams to assist in transforming them into more powerful multi-level Baumkuchen models.

### 2.3. Multi-Level Diagrams

**Figure 5:**
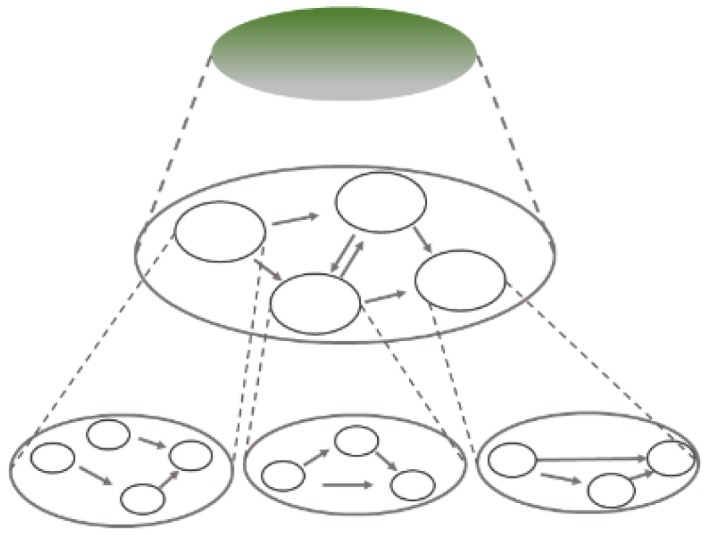
Schematic representation of a multi-level mechanism.

Combining insights about producing and underlying mechanisms enables researchers to construct nested multi-level mechanistic explanations, as illustrated in Figure 5. The interactions between mechanistic components can be interpreted as producing mechanisms within an underlying mechanism. At the same time, any component in an underlying mechanism can itself be analysed as featuring an underlying mechanism which has components which can be mechanistically analysed further; and so on. Eventually, the mechanism “bottoms out” and it is no longer a black box, Figure 5 [3].

This is the basic rationale we adopt in the discussion which follows. Our starting point is to recognize that pharmacokinetic mechanisms usually describe causal chains of production, while pharmacodynamics tend to focus on mechanisms of action underlying the in vivo effects of a drug. As such pharmacodynamic schemes cross different levels of investigation and, they should be represented by a multi-level diagram incorporating productive aspects at various levels, similar to the one in Figure 5. We call the resulting models *Baumkuchen models*, inspired by the layered structure of the famous Austrian Baumkuchen, rather than multi-level mechanisms. As mentioned before, choosing a new term highlights that we do not commit to the idea that the interlevel relations in our diagrams will strictly conform to mechanist definitions of interlevel relations as local part-whole relations [7]. We aim to explicitly leave room for less precisely defined hierarchical relations. Still, as with prototypical multi-level mechanisms, disentangling different kinds of mechanisms may assist practitioners in (i) gaining a better conception of the processes “responsible for” different phenomena within a research domain, (ii) understanding how these phenomena are linked with one another, and (iii) disambiguating between the different relations depicted by arrows in traditional flat pharmacological schemes. We propose that Baumkuchen models provide an amenable, alternative, practical, and more powerful tool to represent “mechanisms of action” in pharmacology as they render different kinds of “being responsible for” relations more explicit. In what follows, we shall present the implications of this claim.

## 3. Mechanisms of Action in Pharmacokinetics and Pharmacodynamics

Pharmacology is the science which focuses on studying the interactions between substances, notably drugs, and living systems [19]. This science is broad and entails—mainly—two intertwined branches: pharmacokinetics and pharmacodynamics. Whilst pharmacokinetics addresses “body–drug” interactions, such as absorption, distribution, metabolism and elimination of a drug, pharmacodynamics is concerned with “drug–body” interactions. Such “drug–body” interactions include the common activities of drugs inside the human body and also the underlying mechanisms of action. Still, both refer to mechanisms of action (MoA) to characterize what is going on, and both fields use traditional mechanistic flat “box and arrow” diagrams. Note that the pharmaceutical notion of MoAs differs from the one employed in other sciences. In systems biology, for instance, a mechanism typically describes the interactions between different parts and processes within a larger overall system. In chemistry, reaction mechanisms may refer to abstract models or hypothetical constructs proposed to explain or predict certain processes or products. Here, we notice that mechanisms may feature different kinds of explananda; still, the commitment to realism is stronger in the case of pharmacology MoAs than those in chemistry.

In *pharmacokinetics*, MoAs describe how a drug enters the body and how it is subsequently distributed, metabolized and excreted, with a focus on the drug itself. Such mechanisms are usually analysed as causal chains of mechanisms and may also be represented by mathematical formulae. They describe the journey and transformation of the drug itself. Spironolactone, for instance, is a potassium sparing diuretic, hence has its indications in handling hypertension and chronic heart failure. Spironolactone reaches its peak plasma concentration after 2.6–4.3 h. It is circulated in the body, with 90% bound to plasma proteins, and undergoes extensive liver metabolism and is then excreted in urine and bile [20,21].

*Pharmacodynamics*, by contrast, employs MoAs, which are predominantly underlying ones, (although there are also instances of producing mechanisms). In its glossary for chemists, the International Union of Pure and Applied Chemistry (IUPAC) defines pharmacodynamics as the “study of pharmacological actions on living systems, including the reactions with and binding to cell constituents, and the biochemical and physiological consequences of these actions.” [22] In other words, pharmacodynamics is an inherently multi-level affair: It is the study of a drug’s MoA and the resultant pharmacological effects at higher levels, i.e., “the biochemical and physiological consequences” of a MoA. As an example for a pharmacodynamic mechanism, consider spironolactone again. Excess levels of mineral corticosteroids result in the retention of sodium and water and, thus, edema. Mechanistically, spironolactone competitively blocks aldosterone from binding to its receptors in the late distal tubule and collecting duct in the nephron, hence reducing sodium reuptake by obstructing the DNA expression of genes for sodium channels and pumps [20,21,23]. Please note how in this simple example we have crossed at least three different levels of complexity; binding to the receptor and obstruction of gene expression can be assigned to the Cell Level, reduction of sodium uptake to the Physiology Level and, further, on avoidance of edema to the Health Level.

In summary, pharmacokinetic MoAs describe productive chains often employing mathematical formulae, e.g., about half-life, whereas pharmacodynamic MoAs describe processes which underlie higher, e.g., organism-level physiological effects of the drug in question. Such higher-level effects are based on “reactions with and binding to cell constituents”, i.e., processes on a lower level responsible for the MoA’s operation. As we shall demonstrate below (Section 5), analysing MoAs as combinations of producing and underlying mechanisms is the most appropriate approach to fathom some of these rather complex interdependences.

This brief exposition begins the discussion we shall elaborate on in the following sections: pharmacologists investigate mechanisms at various different levels. Additionally, despite the fact that the common-place representation for MoAs is non-hierarchical, we already had a glimpse at the multi-level picture hidden underneath. The key to transforming flat pharmacological diagrams to multi-level ones will be, as we shall see in the next section, to recognise that the arrows contained in traditional “box and arrow” diagrams represent different kinds of “being responsible for” relations. Once we disentangle these different arrows, a multi-level representation of pharmacological MoAs as consisting of a variety of inter-connected producing and underlying mechanisms results almost naturally.

## 4. Arrows

**Figure 6:**
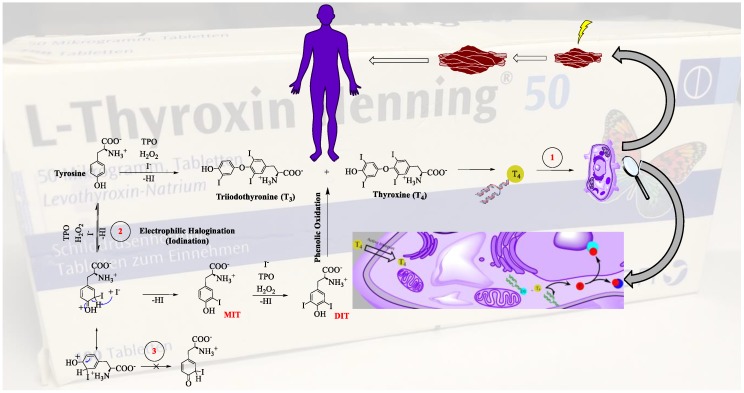
The biosynthesis of thyroid hormones and their impact on healthy muscle growth [5,23,26,27]. This scheme is adapted from two standard pharmacology textbooks, namely, Brenner and Stevens’ Pharmacology 5th edition p. 368 and Basic & Clinical Pharmacology 14th edition p. 688, and purely for illustration purposes, has been expanded to include some additional formulae and conversions.

Common pharmacological schemes and diagrams condense high complexity in two-dimensional images containing lots of boxes and arrows. While boxes represent mechanistic components, and arrows are suggestive of causal relations, this simplified reading is often misleading. To illustrate this point, we now consider the biosynthesis and subsequent activity of the thyroid hormones triiodothyronine (T3) and thyroxine (T4) in the healthy human thyroid gland. This scheme is depicted in Figure 6, which may be found in this or similar forms in most contemporary textbooks of biochemistry or pharmacy. Here, we can easily identify different types of arrows representing producing and underlying mechanisms along with other relations such as time, transfer, modificatory influences, etc. For the time being, we shall focus on how to identify and distinguish arrows indicating producing and underlying relations in MoAs.

### 4.1. The Straight Black Arrow

The most common arrow found in pharmacological diagrams is the straight arrow. It occurs in lots of variations: unidirectional, bidirectional, as a solid line (as conventional in chemistry) or as a block arrow (as conventional in biochemistry and physiology). Such arrows suggest some kind of causality and often carry additional information above, below, or inside. Figure 6 reflects this extensive use of such different arrows. Here, the block arrows designate some kind of connection between biochemical and physiological systems, such as the thyroid gland and the muscles [24]. The assumed causality here is complicated, since the thyroid gland is obviously not “causing” the muscles; rather, processes within the thyroid gland act as difference-makers for processes within the muscular system (arrow tagged with 1 in Figure 6) [25].

More precisely, the arrow implicitly tells us that products produced within the thyroid gland circulate to the muscles, where they influence or control muscle mass. As T3 and T4 are the messengers between these two organs, they should be correctly placed inside the arrow or next to it. Interestingly, the interpretation of this type of arrow is in line with the notion of “systems” in biology, as mentioned earlier in Section 3.

In chemistry, the straight black arrow differs as it connects educts of a chemical reaction with the respective products. This arrow describes a causal relation between chemical compounds, either unidirectional or in equilibrium, and connects chemical symbols which usually—although not always—stand for elements, ions, molecules, etc. In Figure 6, such arrows connect the representations of the educts tyrosine and iodine with the ones for T3 and T4, and also contain some information about the reaction conditions and yields placed next to them. These arrows form a central part of the repertoire of chemical language [28]. As such, their deployment follows a certain syntax and they represent chemical substances and observable events.

In addition to simple straight arrows, there are also arrows pointing at products which may be expected on a theoretical basis. For a number of reasons, though, the anticipated product(s) may only be obtained in the laboratory. The “crossed out” straight arrow highlights that the substance in question is not formed under the reaction conditions employed. Such a special case of a “crossed out” straight, producing arrow is tagged with 3 in Figure 6, where a possible or anticipated product is not obtained in the laboratory, often for a number of possible reasons. Chemists tend to point such a producing arrow at a non-product, perhaps to underline an element of surprise or the possibility that someone else may be more successful later on.

### 4.2. Arrows in Disguise and in Transition

It should also be noted that some of the straight arrows in Figure 6 may point to the “underlying chemical reaction mechanism” as discussed before in Section 3. These straight arrows hardly differ from the ones connecting educts with products. Nonetheless, as part of the “underlying mechanism”, they point to a different level and may also point at intermediates, excited states, transition states, and other chemical structures. These entities may or may not represent tangible substances, as depicted on the bottom left of Figure 6. Indeed, arrows connecting representations of compounds with transition states, i.e., states which do not represent any identifiable components in mechanisms, are problematic. Transition states are often entirely hypothetical and cannot be decomposed mechanistically. Neither can they be associated with any specific chemical compound or mechanistic entity. In Figure 6, a case in point is the transition state following tyrosine to produce monoiodothyrosine (MIT) (arrow tagged with 2). This instance illustrates how a chemical reaction proceeds from the educts to certain intermediate transition state(s) and then to the products. In our example, the direct arrow from tyrosine and iodine to T3 and T4 should thus be read as a producing relation. The arrow passing via the intermediate and transition state, by contrast, depicts the underlying chemical mechanism. These representations are introduced in such schemes to explain certain reactions and reaction pathways. They also assist in determining the course and direction of a chemical reaction and try to explain why some products are obtained, whilst others are not. As just mentioned (and discussed further in Section 5), arrows pointing into transition states frequently cross into the underlying mechanism of the reaction and hence also into a different level within a multi-level scheme.

**Figure 7:**
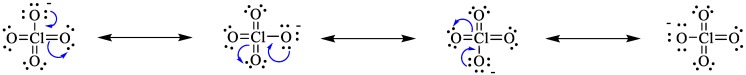
Resonance structures of perchlorate (ClO_4_^-^) connected by arrows indicating mesomerism. The hooked arrows point towards “delocalized” electron pairs which are “moving” in the representation to generate four distinct structures. In contrast, this “movement” cannot be observed experimentally, as there is only one perchlorate molecule. The hooked arrows serve solely the purpose of explanation and to connect the four resonance structures required to describe perchlorate, its properties, acidity of perchloric acid, spectroscopy and reactivity fully.

Interestingly, the chemical reaction sequence in Figure 6 contains several different types of apparently similar arrows. Early on, students of biochemistry and related fields learn to distinguish straight black arrows from the rather unusual double-pointed arrows (indicating mesomerism). Such arrows do neither indicate causal or producing nor underlying relationships. The double-pointed straight arrows shown in Figure 7, for instance, indicate that perchlorate has four contributing structures resulting from an evenly delocalized electron pair [29].

As we dig deeper, another kind of arrow appears in Figure 6 and Figure 7, hidden inside or next to chemical structures: The hooked arrow. It denotes the “movement” of an electron (single headed hooked arrow) or electron pair (double headed hooked arrow), either inside a given molecule or between molecules. It is found, for instance, in mesomeric structures and also in the so-called reaction mechanisms. At closer inspection, the hooked arrow provides information about the underlying—electronic—mechanism of the underlying chemical reaction mechanism in a nested situation similar to the one described in Figure 5. Indeed, the arrows connecting mesomeric structures and transition states may be seen as parts within an underlying chemical reaction mechanism proceeding from educts to products, viz. they are located at the same layer. By contrast, the moving electrons indicated by the hooked arrow appear to be underlying this mechanisms, viz. they are located at the next lower layer and will be described in a lower-level mechanistic explanation.

As we shall explain in the following section, the mechanism which underlies the underlying mechanism of the chemical reaction is considered to reside at the lowest level in the case at hand. It is important to note, though, that this reflects a pragmatic choice of the “bottoming-out” point for the discipline of pharmacy rather than a metaphysical commitment; we find it applied in many illustrations in the field (see, e.g., Figure 6). Still, the scheme can in principle be expanded as we move further down the mechanistic hierarchy, possibly all the way to protons, neutrons, electrons and even quarks.

## 5. Constructing Baumkuchen (Spite Cake) Models

Our discussion so far has revealed that traditional “box and arrow” diagrams in pharmacology may be composed of a medley of different kinds of mechanisms contained within a “flat”, i.e., non-hierarchical, scheme, describing certain (bio-)chemical processes which actually cross different levels of investigation. In order to reveal—at least some of—the ambiguities which may result from such a collapse of a multi-level hierarchy into flat schemes, we need to (a) distinguish between different levels or layers of analysis (e.g., from electron movements in molecules at the “Resonance Level” all the way up to medical conditions at the “Health Level”), (b) distinguish between different kinds of mechanisms (especially producing and underlying ones), and (c) disambiguate between different kinds of arrows and which kinds of “being responsible for” relations they depict. If this task is taken seriously, a multi-layer Baumkuchen model can be constructed out of non-hierarchical pharmacological diagrams. Such a representation can be achieved by drawing on our discussion above to provide a practical and powerful tool to represent MoAs in pharmacology.

**Figure 8:**
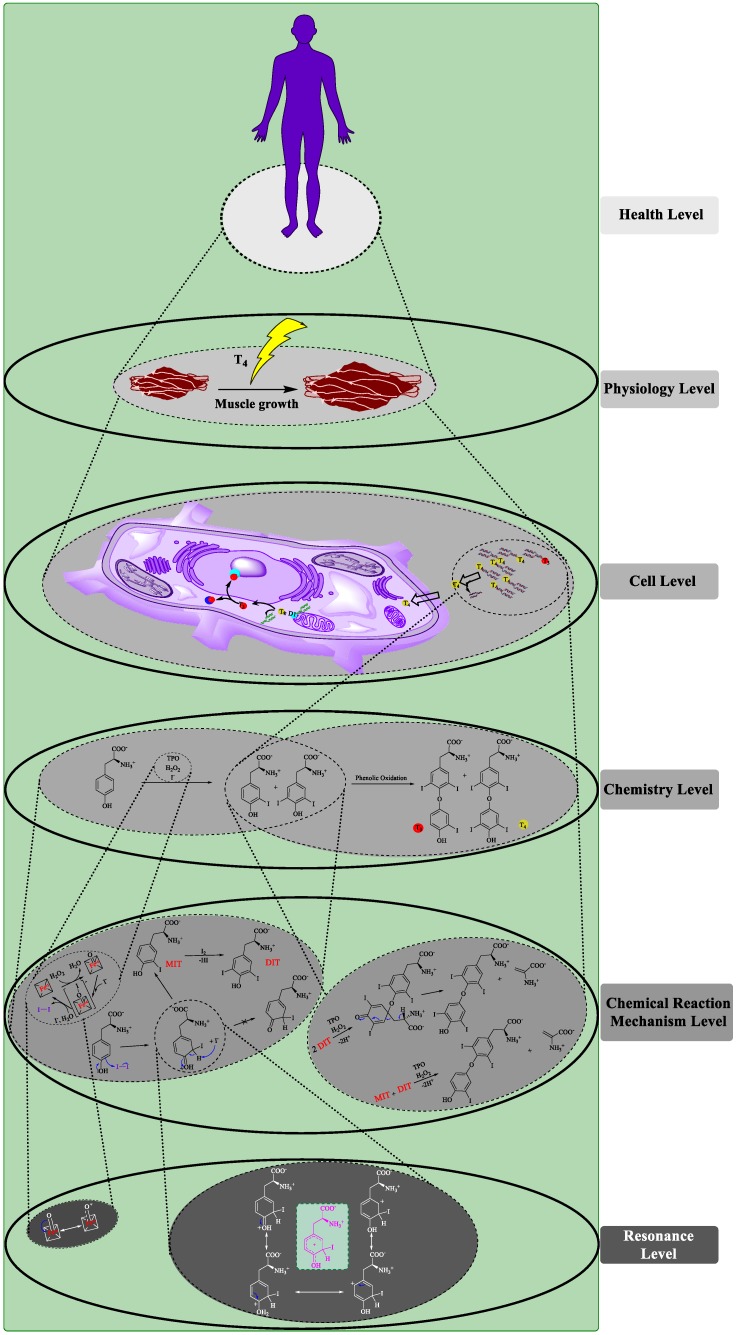
The Baumkuchen model of the initially “flat” biochemical scheme depicted in Figure 6. Here, the different layers are clearly separated. Producing mechanisms within layers are shown in straight arrows, and underlying mechanisms depicted with cones, similar to the model in Figure 5. Please note that the Baumkuchen model avoids arrows between layers. If shown, these arrows may serve as heuristic tools rather than indicators of interlevel causation.

What does this look like for our case study? Figure 8 provides a Baumkuchen model of the thyroid hormone formation and its action in the human body [5,23,26,27,30,31,32,33]. As such, Figure 8 is effectively a revamped and more precise version of Figure 6. Different layers—in this case coinciding with different scientific disciplines—are separated clearly. Within each layer, producing mechanisms are at work. Different layers are connected by underlying mechanisms. The arrow between MIT and T3 and T4 at the “Chemistry Level”, for instance, is clearly pointing to a causal mechanism, whilst the top of the cone stemming from T3 and T4 underlies the formation of their biochemical complex with Thyroxine-binding globulin (TBG) in the bloodstream. The representation in Figure 8 also disambiguates effectively the different “being responsible for” relations, which most of the straight black arrows in Figure 6 denoted: arrows representing producing relations are located within any given layer while arrows indicating constitutive relations characteristic of underlying mechanisms are replaced by cones. These interlevel relations are not usually considered causal. In fact, some proponents of the mechanistic view, e.g., Craver and Bechtel [34], argue that there is no interlevel causation. In contrast, we suspect that denying the possibility of interlevel (interlayer) causal relations is premature; and hence we leave room for it in our Baumkuchen models and will address this issue in a subsequent manuscript. After all, an electron from the “Chemistry Level” may directly (causally) interfere with a process at the “Cell Level”. Still, this interference may be mediated by a series of constitutive relations through the Baumkuchen model. Yet, strictly speaking, all the cones depict is an empirically observed interlevel difference-making. And this may indicate multiple things: a causal link (with or without intermediates), a constitutive relation, or even that another (yet unknown) underlying mechanism needs to be identified.

As we look closer at the different layers within the Baumkuchen model in Figure 8, we note that the lowest level we need in this kind of scheme is the one of resonance or electron pair movements. As discussed already, these structures, with their moving electrons and hooked arrows, form the underlying electronic mechanism(s) of the chemical reaction as it proceeds stepwise from educts to products, and, if necessary, also via transition states and mesomeric resonance structures. All of these entities and activities are located at the bottoming-out level of the scheme, providing the information needed to explain the reaction mechanisms which underlie transition states and mesomeric structures occurring further up in the hierarchy.

These constructs, together with reaction intermediates, form the second level of the Baumkuchen, which is home to the underlying reaction mechanism explaining the chemical reaction itself, and including the intermediate MIT. This chemical reaction, in our case proceeding from tyrosine and iodine to T3 and T4, resides on the third level, which we have referred to as “Chemistry Level” before. Curiously, in physiology, the chemical reaction itself is often considered as “underlying” and tends to be referred to as the “mechanism of biological action” or “mode of action”. Interestingly, the choice of the bottom level may differ according to discipline. While a chemist considers a chemical reaction to be on top of the cone, towering above the reaction mechanism and resonance levels, the physiologist, in contrast, will build the cone on the chemical reaction itself. Subsequently, the pharmacological mechanistic analysis of how thyroid gland hormones act in the human body bottoms out at the transition from tyrosine and iodine to T3 and T4. This ignores the fact that the chemical conversion process in question can also be considered as a mechanism on its own. The subsequent layers of the cake are rich in biochemistry. Here, chemical interactions, for instance of T3 and T4 with their respective receptors, result in physiological changes, such as muscle growth. Higher up, this layer of physiological processes provides the underlying mechanism(s) for health and disease, in more general terms.

The Baumkuchen model does have several advantages over traditional non-hierarchical models. Most importantly perhaps, it is richer in content and disambiguates different dependency relations based on careful analysis. It allows researchers to zoom into individual layers and to focus in on specific aspects of the overall state of affairs. Still, it is far from perfect: several “unidentified” arrows and relations remain, not all entities and activities can be clearly located at specific levels, it is not clear what adequate levels are in any given case, etc. Besides, we have not discussed how to incorporate regulatory maintaining mechanisms into our Baumkuchen picture. While a detailed discussion of this issue asks for a separate publication, we guess that—at least in principle—it should be possible to analyse maintaining mechanisms as continuously operating underlying or producing mechanisms or a combination thereof (see [6] for discussion).

## 6. Conclusions

We have demonstrated that careful analysis of ambiguous “flat” pharmacological “box and arrow” schemes may benefit all parties involved. Importing the concepts of producing and underlying mechanisms from new mechanical philosophy of science to pharmacology has allowed us to propose a new type of diagram for pharmacologists to employ in practice. The Baumkuchen model provides a powerful alternative to traditional pharmacological diagrams. It has all the representational resources of traditional “flat” diagrams whilst adding information about interlevel relations and disambiguating between the different relations represented by arrows in traditional “box and arrow” schemes. Importantly, the Baumkuchen model is easy to construct with familiar tools and resources and subsequently may be expanded to interactive virtual electronic versions of such multi-layered schemes. This is indeed the case with conventional flat diagrams in the pharmacology textbooks utilized in this manuscript [5,23]. Nowadays, such books are accompanied with a CD or an online account activation code where the user is given the chance to glance closer onto the schemes in an interactive manner. Building on its “flat” predecessor, however, the Baumkuchen model would certainly offer a change of representation which allows researchers to effectively communicate more information with well-established tools. 

That said, let us remind our readers that we are not aiming to draw any strong metaphysical conclusions. Whilst we do share a certain realist commitment, we are certainly aware that empirical evidence may underdetermine which Baumkuchen model is actually correct or most realistic. Still, even hypothetical or inaccurate multi-level models can progress research. At the very least, they may offer hypotheses as to how pharmacological mechanisms work together to give rise to complex phenomena, such as the action of thyroid gland hormones in the human body. As such, they potentially offer deeper insights for pharmacology research than traditional “box and arrow” schemes can possibly offer. Therefore, we submit that constructing multi-level Baumkuchen models will help pharmacologists to gain deeper insights into how various MoAs are linked and also to guide future research.
